# A computational analysis of transcriptional profiles from CD8(+) T lymphocytes reveals potential mechanisms of HIV/AIDS control and progression

**DOI:** 10.1016/j.csbj.2021.04.056

**Published:** 2021-04-25

**Authors:** Sergey Ivanov, Dmitry Filimonov, Olga Tarasova

**Affiliations:** a Department of Bioinformatics, Institute of Biomedical Chemistry, Moscow, Russia; b Department of Bioinformatics, Pirogov Russian National Research Medical University, Moscow, Russia

**Keywords:** Human immunodeficiency virus, Elite controllers, CD8^+^ T lymphocytes, Gene expression, Cluster analysis, Master regulators

## Abstract

Cytotoxic and noncytotoxic CD8^+^ T lymphocyte responses are essential for the control of HIV infection. Understanding the mechanisms underlying HIV control in elite controllers (ECs), which maintain undetectable viral load in the absence of antiretroviral therapy, may facilitate the development of new effective therapeutic strategies.

We developed an original pipeline for an analysis of the transcriptional profiles of CD8^+^ cells from ECs, treated and untreated progressors. Hierarchical cluster analysis of CD8^+^ cells’ transcription profiles allowed us to identify five distinct groups (EC groups 1–5) of ECs. The transcriptional profiles of EC group 1 were opposite to those of groups 2–4 and similar to those of the treated progressors, which can be associated with residual activation and dysfunction of CD8^+^ T-lymphocytes. The profiles of groups 2–4 were associated with different numbers of differentially expressed genes compared to healthy controls, but the corresponding genes shared the same cellular processes. These three groups were associated with increased metabolism, survival, proliferation, and the absence of an “exhausted” phenotype, compared to both untreated progressors and healthy controls. The CD8^+^ lymphocytes from these groups of ECs may contribute to the control under HIV replication and slower disease progression. The EC group 5 was indistinguishable from normal.

Application of master regulator analysis allowed us to identify 22 receptors, including interferon-gamma, interleukin-2, and androgen receptors, which may be responsible for the observed expression changes and the functional states of CD8^+^ cells from ECs. These receptors can be considered potential targets of therapeutic intervention, which may decelerate disease progression.

## Highlights

• Distinct groups of HIV elite controllers’ transcription profiles from CD8^+^ cells are exist.
• Three groups associated with increased metabolism, survival, and proliferation of CD8^+^ cells.
• One group may be associated with residual activation and dysfunction of CD8^+^ cells.
• Elite controllers’ transcription is dissimilar to treated and untreated progressors.
• Cytokine and androgen receptors on CD8^+^ cells may be essential for HIV control.

## Graphical abstract

**Figure:**
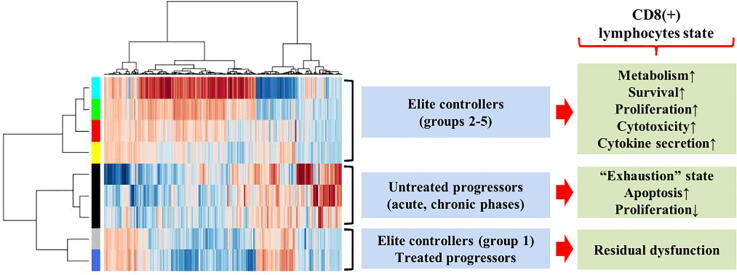

## 1 Introduction

Human immunodeficiency virus (HIV) infection remains one of the most significant challenges facing humankind. Approximately 38 million people were living with HIV, and 690 thousand died by the end of 2019 (https://www.who.int/news-room/fact-sheets/detail/hiv-aids). Early administration of combined antiretroviral therapy (cART) enables HIV infection to switch to a chronic form and increases the lifespan of patients to those seen in uninfected people; however, irregular use of cART and adverse effects caused by particular drugs and their toxicity significantly hinder the efficiency of this approach [1], [2], [3]. Moreover, since the presence of latent HIV infection, cART must be administered throughout life because interruption of therapy leads to viral rebound and disease progression [4], [5], [6]. Thus, new approaches to treat HIV infection are being developed [5], [7], [8], [9], [10]. One of the most promising approaches is developing therapeutic and preventive vaccines; however, no effective vaccines exist at present [7]. This lack of a vaccine can be explained by the fact that people do not develop natural, protective immunity to HIV infection, whereas almost all successful vaccines were created for diseases for which natural immunity exists [9]. Since some vaccine candidates allow moderate protection from HIV, a successful vaccine may potentially be developed; however, to this end, a more profound understanding of the interaction between HIV and the immune system is required.

HIV infects CD4^+^ T helper cells, as well as monocytes and macrophages and various kinds of dendritic and epithelial cells [11]. HIV infection causes innate and later adaptive immune responses, where the latter includes both cellular and humoral components. The formation of anti-HIV antibodies may be essential for control under viremia [12]. On the other hand, the CD8^+^ cellular response seems to have a significant role [13], [14]. The cellular response causes a decline in viral load in the acute phase of infection but cannot eliminate the virus from the organism because a high mutation rate during replication allows HIV to escape from the immune response. Persistent infection causes chronic activation of HIV-specific CD4^+^ and CD8^+^ cells, which leads to their apoptosis [15], [16]. A phenomenon called “bystander activation” was described in chronically infected patients, in which significant numbers of CD4^+^ and CD8^+^ non-HIV-specific T lymphocytes are activated in a T-cell receptor-independent and cytokine-dependent manner that leads to their apoptosis [13], [17], [18], [19], [20]. Chronic infection also causes the dysfunction or “exhaustion” of CD8^+^ lymphocytes, which is characterized by increased expression of inhibitory immune checkpoints PD-1, Lag-3, and Tim-3 on the surface of cells and decreased ability of CD8^+^ cells to proliferate, secrete cytokines, and induce cytotoxicity [21]. The high mutation rate of HIV, immune exhaustion and the loss of CD4^+^ and CD8^+^ cells make further immune responses ineffective with subsequent progression to AIDS [22]. However, the time from infection to the development of AIDS can vary significantly among patients. Most infected patients, called progressors, usually develop AIDS after 8–10 years, but a small group of people, known as long-term non-progressors (LTNPs), remains asymptomatic for more than ten years and are characterized by high CD4^+^ cell counts (more than 500 cells/ml) [16], [23], [24], [25]. Another related group of patients, called elite controllers (ECs), demonstrates the best control of viral replication with a viral load less than 50 copies/ml for at least one year [16], [23], [24], [25]. Little is known about the mechanisms of viral control in ECs, but it seems that host and viral factors, as well as various cell types, may be involved [13], [16], [22], [23], [24], [25], [26], [27], [28]. Among these mechanisms, the cytotoxic and noncytotoxic responses of CD8^+^ lymphocytes seem to play a major role [13], [16], [22], [23], [24], [25], [26], [28]. Naive CD8^+^ T lymphocytes recognize MHC-I restricted HIV antigens presented by dendritic cells in lymph nodes that cause their activation, proliferation, and differentiation into cytotoxic T lymphocytes. After that step, HIV-specific CD8^+^ lymphocytes are capable of recognizing viral antigens in complex with MHC-I on the surface of infected cells and cause their apoptosis by secretion of perforin and granzyme B, as well as by FasL–Fas interaction [13], [16]. The virus enters the cell using the CD4 receptor and various coreceptors, including C-X-C chemokine receptor type 4 (CXCR4) and C–C chemokine receptor type 5 (CCR5) [29]. Thus, CD8^+^ lymphocytes can also exhibit noncytotoxic anti-HIV functions through the secretion of chemokines that compete with HIV particles for the corresponding co-receptors [23]. CD8^+^ lymphocytes from ECs have T-cell receptors capable of broader cross-recognition of mutated epitopes from HIV gag antigens, which may be a consequence of the presence of HLA-I polymorphisms, e.g., HLA-B*57 and B*27, that influence the selection of T cell clones in the thymus [22], [23], [26], [30]. CD8^+^ lymphocytes from ECs do not demonstrate an “exhaustion” state and have preserved functions related to cytotoxic and noncytotoxic responses [22], [23], [26], [28], including the ability to degranulate and secrete cytokines with anti-HIV effects, such as MIP-1α/β, RANTES, IFN-γ, TNF-α, and IL-2. They also have a higher proliferation rate and low level of apoptosis compared to progressors.

Investigation of CD8-related mechanisms of viral control in ECs is essential because mimicking similar responses in chronically infected progressors may lead to the functional remission of HIV infection [31]. To date, most of the studies related to the investigation of mechanisms of HIV suppression in ECs have focused on particular molecules or pathways, whereas analysis of HIV-related genome-wide OMICs data provides an opportunity to reveal novel mechanisms that were not known or previously hypothesized [32], [33], [34]. Transcriptomics studies are the most frequent in HIV research and include those investigating CD8^+^ lymphocytes from ECs and LTNPs [35], [36], [37], [38]. Most of the corresponding studies were focused on total CD8^+^ lymphocytes, rather than HIV-specific lymphocytes [35], [37], [38]. This focus is important because non-HIV-specific CD8^+^ and CD4^+^ lymphocytes are involved in the pathogenesis of disorder by the “bystander activation” effect (see above), which leads to their dysfunction and apoptosis [13], [17], [18], [19]. On the other hand, non-HIV-specific CD8^+^ cells may still contribute to the control under HIV replication in ECs, e.g., by secretion of cytokines with anti-HIV effects [18]. To identify specific HIV control mechanisms, the comparison of gene transcription in CD8^+^ lymphocytes from ECs to both progressors and healthy controls is required. For instance, Hyrcza and colleagues compared transcription profiles from total CD4^+^ and CD8^+^ lymphocytes between LTNPs, progressors (acute and chronic phases), and uninfected people [35]. They found differentially expressed genes (DEGs) between LTNPs and progressors in both the acute and chronic phases but did not find differences between LTNPs and healthy controls. This can potentially be explained by the fact that CD8^+^ from ECs and LTNPs have heterogeneous transcription profiles, and some of them are indistinguishable from healthy controls. Chowdhury and colleagues applied cluster analysis to CD8^+^ cell transcriptional profiles from 51 ECs and found five distinct groups [38]. Some of the groups were distinguishable from both control samples and samples from cART-treated patients. The authors found that the pathways governed by mTOR and eIF2 proteins are potentially the most important for the functions of CD8^+^ lymphocytes in ECs and that these pathways are dominant in three out of five EC groups.

In the present study, we performed a comprehensive analysis of the transcription profiles of CD8^+^ lymphocytes from ECs using an original pipeline. In contrast to earlier studies [35], [37], [38], where transcription profiles from ECs were compared to profiles from either cART-treated or untreated progressors, we compared gene expression between ECs and both categories of progressors as well as uninfected individuals. While previous researches on CD8^+^ transcriptome [35], [37], [38] were focused on few pathways and proteins, e.g., mTOR and eIF2 pathways, or interferon-stimulated genes, we carried out a thorough comparison of investigated ECs’ and progressors’ groups using large amounts of pathways and small groups of functionally related genes. Most importantly, we performed an analysis of master regulators (MRs), which are the proteins at the top of the signaling network regulating the expression of DEGs observed in ECs.

The developed pipeline includes the following steps: (1) identification of distinct groups of ECs based on the corresponding transcription profiles using hierarchical clustering; (2) identification of differential expression for each EC group, cART-treated and untreated progressors compared to uninfected controls; (3) comparison of obtained DEGs between ECs and cART-treated and untreated progressors; (4) identification and comparison of differentially regulated pathways and small groups of functionally related genes in ECs and progressors; and (5) identification of receptors, which are potential MRs and may be responsible for observed gene expression changes in EC groups compared to healthy controls. The application of the pipeline to CD8^+^ lymphocytes’ transcription profiles allows us to identify heterogeneous groups of ECs with potentially different mechanisms of HIV control, describe the functional state of CD8^+^ cells for each group at the level of genes and pathways, estimate similarity and/or differences of EC groups to cART-treated and untreated progressors, and reveal receptors for cytokines and hormones, which may be responsible for observed transcription profiles and, as a result, preserved or enhanced functions and survival of CD8^+^ lymphocytes.

## 2 Results

### 2.1 Identification of EC groups based on CD8^+^ cell transcription profiles

We analyzed all available datasets from the Gene Expression Omnibus (GEO) (https://www.ncbi.nlm.nih.gov/geo) belonging to ECs and LTNPs as well as uninfected individuals. We observed that datasets with the following GEO IDs: GSE87620, GSE6740, GSE28128 include CD8^+^ cells transcription data from ECs and LNTPs. The dataset GSE87620, created by Chowdhury and colleagues [38], includes samples from 51 ECs, 32 cART-treated patients, and ten uninfected individuals. Both GSE6740 and GSE28128 datasets contain data on five LTNPs, and the GSE28128 dataset also contains data on eight ECs. GSE6740 and GSE28128 datasets contain data mostly on LTNPs, which may differ by mechanisms and CD8^+^ transcription profiles from ECs [39]. ECs are defined mainly based on low viral load, whereas LTNPs are mainly based on slow disease progression (decrease in CD4^+^ cell counts) [40]. The LTNPs may have comparatively high viral load but slow disease progression as individuals from GSE6740 and GSE28128 datasets. Therefore, we may conclude that due to low viral load ECs may be non-progressors if they are characterized by low disease progression; but we cannot regard LTNPs as ECs at the same time. Therefore, these two groups should be analyzed separately. It is important to note that the number of samples (by five samples from GSE6740 and GSE28128) is too low to perform the clustering analysis for LTNPs’ samples.

Then, transcription profiles from three datasets (GSE87620, GSE6740, and GSE28128) were measured on three different microarray platforms: Illumina HumanHT-12 V4.0 expression bead chip (GSE87620), Illumina HumanWG-6 v3.0 expression bead chip (GSE28128), and Affymetrix Human Genome U133A Array (GSE6740). To merge all samples from three datasets, cross-platform normalization is required. To do it, the same or similar microarray platforms are needed. In our case, two very different platforms (Illumina and Affymetrix) were used. Moreover, to merge and normalize data, the influence of two factors on gene expression must be evaluated: the effect of disease (e.g., ECs vs. healthy people) and batch effect (e.g., the effect of microarray platform). For this analysis, each dataset must contain transcriptional data from both ECs and healthy people [41]. Unfortunately, the GSE28128 dataset, based on Illumina microarray as the GSE87620 dataset, does not contain samples from healthy people. I It makes corresponding analysis impossible. Since the two problems mentioned above cannot be outperformed, we have chosen the dataset GSE87620 for further analysis because it (i) contains the largest amounts of samples from ECs obtained in similar experimental conditions, including microarray platform, and (ii) includes data on samples of CD8^+^ lymphocytes from healthy uninfected individuals, which is essential for the analysis.

**Fig. 1:**
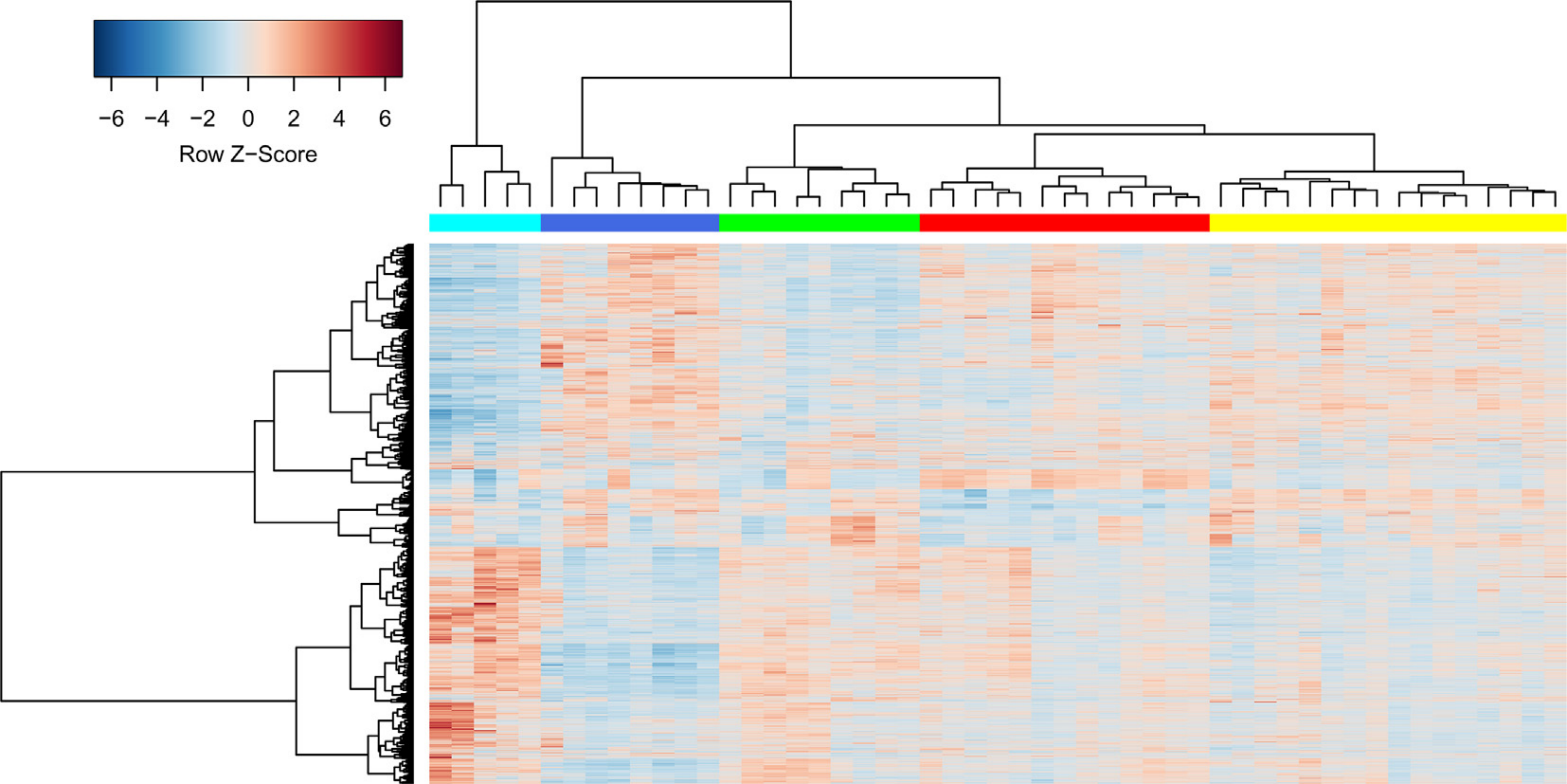
**Heatmap demonstrating clustering results of CD8^+^transcription profiles from ECs.** The rows in the heatmap are genes; the columns are samples. The blue, cyan, green, red, and yellow colors of columns represent EC groups 1–5 (Table 1). Row Z-Score is the number of standard deviations by which the value of gene expression in particular sample is above or below the mean value of all samples. (For interpretation of the references to color in this figure legend, the reader is referred to the web version of this article.)

We performed hierarchical clustering of 51 ECs’ samples from the GSE87620 dataset in the space of 7113 genes with the highest variance of expression values across samples (see Materials and Methods) and found five distinct clusters (Fig. 1) (see also Table S1).

Fig. 1 shows that transcription profiles from ECs are heterogeneous, and some of the revealed groups have opposite profiles (“cyan” and “blue” groups). These results are in agreement with earlier study by Chowdhury and colleagues [38]. In accordance to this study, the heterogeneity of CD8^+^ T cell transcription profiles from 51 ECs cannot be explained by differences in age, gender, race and ethnicity of individuals, as well as presence of protective alleles HLA-B*57/B*27/B*5801, the duration of HIV infection, viral load, the ratio of CD4^+^ / CD8^+^ cells, total CD4^+^ counts, the neutralizing breadth of HIV-specific antibodies, and the breadth and magnitude of HIV-specific cytotoxic response [38]. Thus, the observed differences in gene transcription may be due to the differences in functional state of CD8^+^ lymphocytes essential for viral control.

To estimate the significance of the obtained EC groups, we performed bootstrap resampling analysis and obtained p-values for each cluster: the higher the p-value, the more probable the existence of a cluster. Two types of p-values were calculated: the AU (Approximately Unbiased) p-value and BP (Bootstrap Probability) value, which were computed by multiscale and normal bootstrap resampling, respectively (see Materials and Methods). Most clusters have significant p-values of more than 0.95, and two clusters have p-values of more than 0.65 (Table 1), which indicates that revealed clusters are relatively stable under perturbations.

**Table 1:** The significance of EC clusters.

**Cluster No**	**Color in heatmap**	**No of samples**	**AU p-value**	**BP p-value**
1	Blue	8	0.99	0.99
2	Cyan	5	0.66	0.65
3	Green	9	0.78	0.65
4	Red	13	0.99	0.98
5	Yellow	16	0.99	0.97

Hereafter, we will refer to EC groups by numbers in order as in Table 1 (EC groups 1–5). The associations between particular samples and their groups are available from Table S1.

### 2.2 Identification of DEGs and their comparison between EC groups and cART-treated and untreated progressors

We identified DEGs between each of the EC groups and healthy controls as well as between cART-treated progressors and controls (Table 2). We also retrieved two other datasets with CD8^+^ cell transcription profiles from untreated progressors and corresponding healthy controls, available in GEO (see Materials and Methods). The first dataset (GEO ID: GSE6740), published by Hyrcza and colleagues, contains data on CD8^+^ lymphocytes from untreated progressors in acute and chronic phases of infection [35]. The second dataset (GEO ID: GSE25669) contains corresponding information from untreated progressors in the acute phase. Since the transcription profiles were measured on different microarrays, we identified DEGs for each dataset separately. Numbers of up- and downregulated genes with log fold change > |0.7| and adjusted P-value less than 0.1 in various groups of ECs and progressors are given in Table 2. The corresponding thresholds were chosen empirically to balance the number of DEGs and statistical significance of differential expression. Only EC groups 2 and 3, as well as untreated progressors, were associated with a high number of DEGs, whereas other groups containing the most samples were only slightly different from healthy controls.

**Table 2:** Numbers of up- and down-regulated genes identified in groups 1–5 of ECs and progressors.

**Groups**	**No of samples**	**Up-regulated**	**Down-regulated**
EC group 1 (blue)	8	4	65
EC group 2 (cyan)	5	1063	1073
EC group 3 (green)	9	263	351
EC group 4 (red)	13	79	16
EC group 5 (yellow)	16	14	17
cART-treated progressors	32	14	86
Untreated progressors (acute phase) 1 (GSE6740)	5	362	303
Untreated progressors (acute phase) 2 (GSE25669)	4	331	479
Untreated progressors (chronic phase) (GSE6740)	5	95	118

**Fig. 2:**
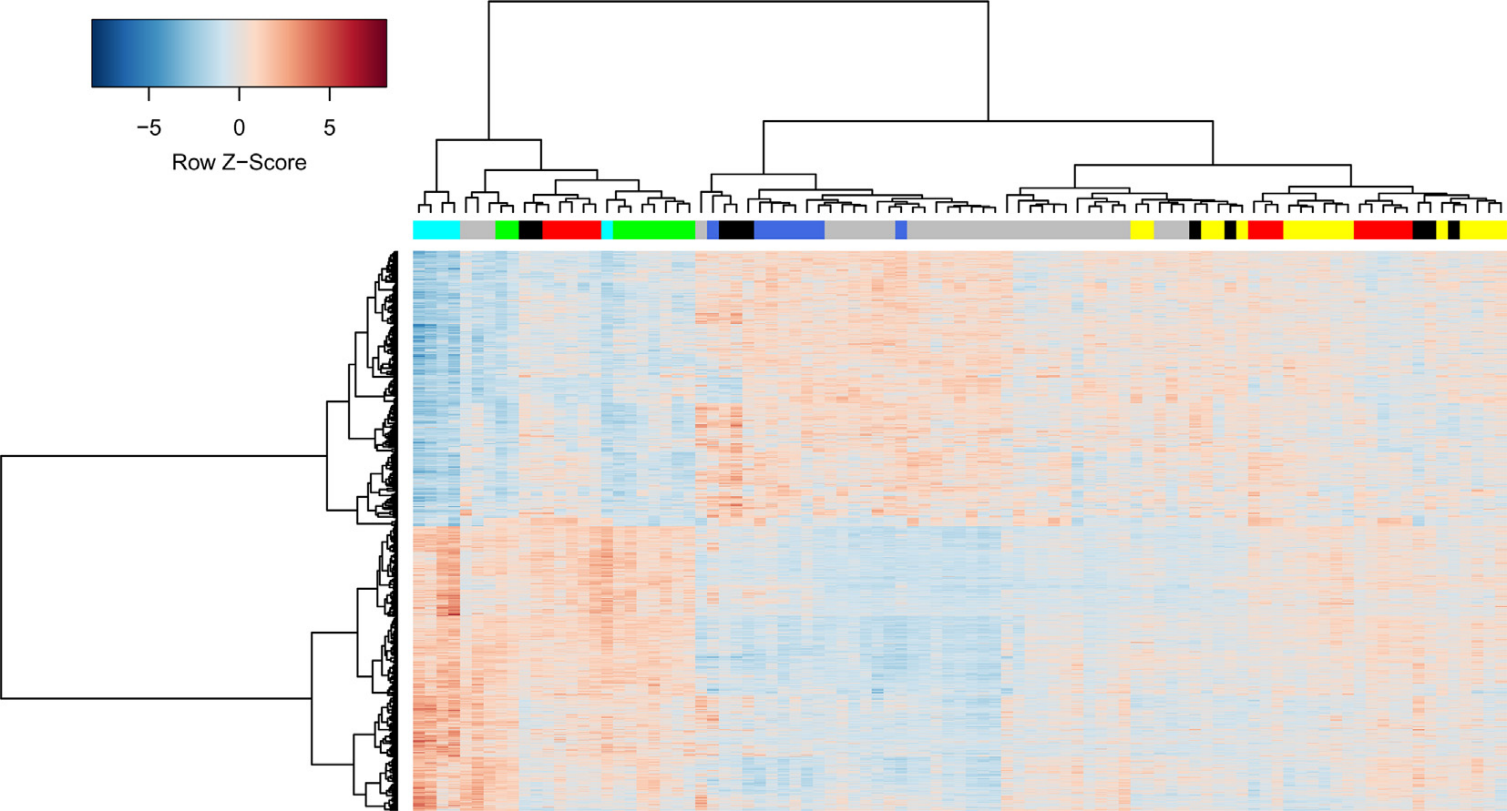
**Comparison of five EC groups with cART-treated progressors and healthy controls.** The rows in the heatmap are genes; the columns are samples. The blue, cyan, green, red, and yellow colors of columns represent EC groups 1–5 (Table 1); grey and black colors represent cART-treated progressors and healthy controls, respectively. Row Z-Score is the number of standard deviations by which the value of gene expression in particular sample is above or below the mean value of all samples. (For interpretation of the references to color in this figure legend, the reader is referred to the web version of this article.)

To compare transcriptional profiles between EC groups together with cART-treated progressors and healthy controls, we performed cluster analysis in the space of genes that were differentially expressed in at least one EC group or cART group compared to the healthy control (Fig. 2).

Analysis of the content of Table 2 and Fig. 2 allows the following conclusions to be drawn. First, all transcription profiles from EC group 5 and some profiles from EC group 4, as well as cART-treated progressors, are indistinguishable from healthy controls (right part of Fig. 2). This finding may indicate that total CD8^+^ lymphocytes from corresponding ECs are potentially not involved in control under viremia. However, it should be noted that the changes in the functional state of CD8^+^ cells from ECs and other HIV-infected people may not be associated with changes in gene transcription but can be related to the changes in the proteome and post-translational modifications [29], [32]. Since some samples from cART-treated progressors are clustered together with samples from healthy individuals, considerable control under viral replication may be achieved in corresponding cART-treated progressors. As samples from cART-treated progressors are divided into two groups on the dendrogram (Fig. 2), we performed bootstrap resampling analysis in a similar manner to the case of ECs but did not find any stable clusters. Second, transcriptional profiles from EC groups 2, 3, and, partially, group 4 (cyan, green, and red color in the left part of Fig. 2) are clearly distinguishable from the healthy control. These groups are similar to each other in terms of transcriptional profiles, but they differ in the magnitude of gene expression changes. For example, EC group 2 was associated with 1063 upregulated genes with log fold change greater than 0.7 and adjusted p-value less than 0.1. However, only 232 of 1063 genes were also upregulated in group 3, and this number increased when the threshold was lowered, e.g., 447 genes were upregulated with log fold change greater than 0.5 and 559 genes with log fold change greater than 0.3. Nevertheless, significant numbers of DEGs are unique for each of the three groups, e.g., 315 of 1063 genes are upregulated in EC group 2 but not upregulated in EC group 3 with any log fold change thresholds at p-value less than 0.05. Similarly, 84 of 263 genes were upregulated in EC group 3 but not upregulated in EC group 4. The same differences were observed for downregulated genes. Third, EC group 1 (blue color) and a significant portion of cART-treated progressors had transcription profiles opposite those in EC groups 2, 3, and 4 (middle part of Fig. 2).

**Fig. 3:**
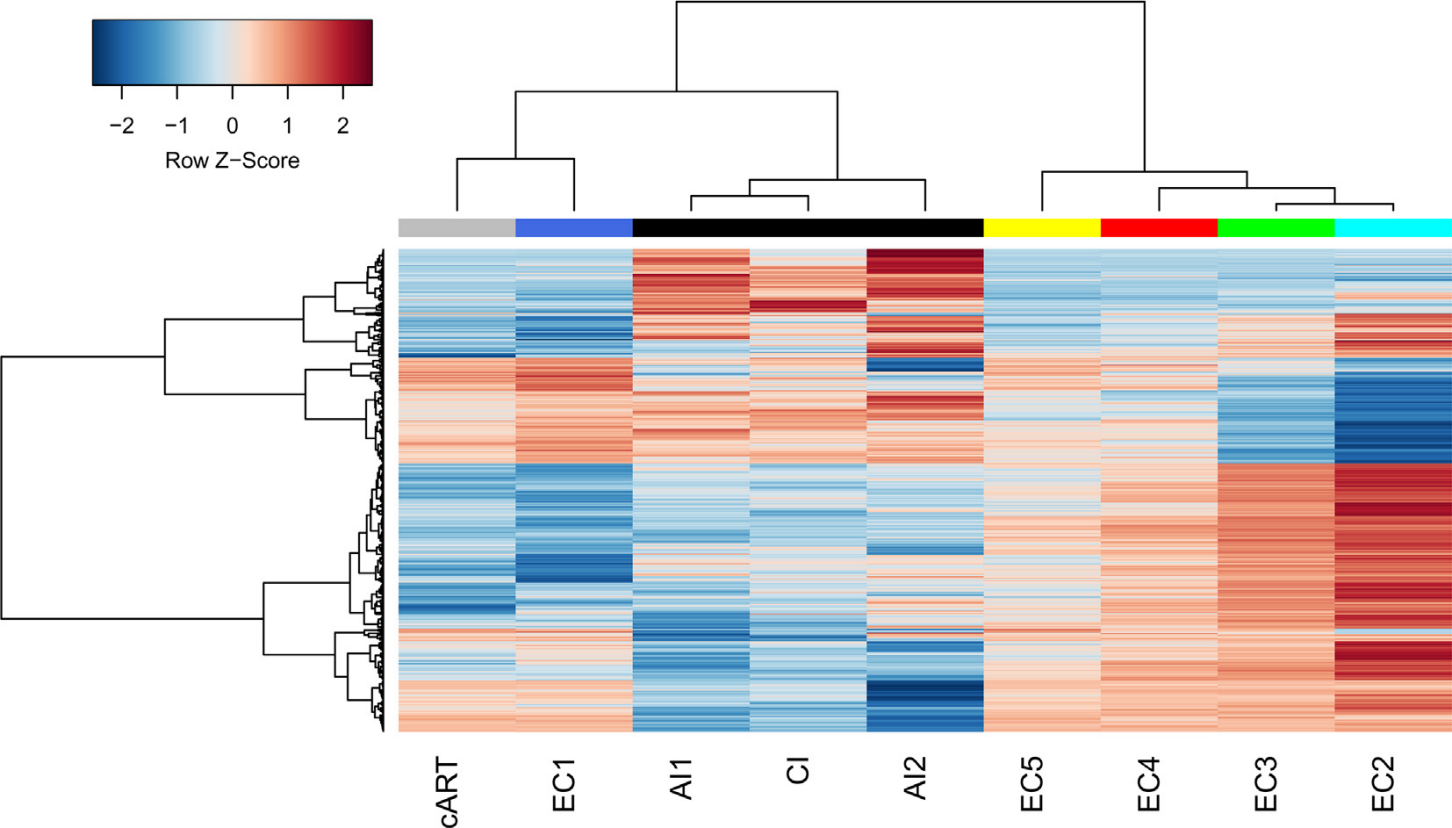
**Comparison of log fold changes from five EC groups, cART-treated and untreated progressors.** The rows in the heatmap are genes; the columns are groups of HIV-infected individuals. The EC groups 1, 2, 3, 4, 5 (EC1-5) are marked by blue, cyan, green, red, and yellow colors. cART-treated progressors (cART) are marked by grey color. Untreated progressors in acute (AI1 and AI2) (GSE6740 and GSE25669 GEO datasets, correspondingly) and chronic (CI) (GSE6740 GEO dataset) phases are marked by black color. Only genes, which were differentially expressed in at least one of the investigated groups (Table 2), were used to create the heatmap. Row Z-Score is the number of standard deviations by which the value of log fold change in particular column is above or below the mean value of all groups. (For interpretation of the references to color in this figure legend, the reader is referred to the web version of this article.)

The transcription profiles from ECs and cART-treated progressors cannot be directly compared with untreated progressors' profiles, since they were measured on different microarray platforms. Instead, we performed a cluster analysis of corresponding log fold changes, calculated by dividing the average expression values in each group to the average expression values in healthy controls (Fig. 3).

Although transcription profiles from untreated progressors in the acute phase (see AI1 and AI2 labels on Fig. 3) were derived from different datasets (GSE6740 and GSE25669) and measured on different platforms (Affymetrix Human Genome U133A Array and Illumina HumanHT-12 V4.0 expression bead chip), the corresponding fold changes are clustered together to confirm the correctness of the approach used. Profiles from different phases of infection (acute and chronic) are also similar (see AI1 and AI2 labels for acute phase and CI label for the chronic phase in Fig. 3). An earlier study by Hyrcza and colleagues did not reveal DEGs between samples of CD8^+^ lymphocytes from acute and chronic phases [31]. Thus, we will further refer to profiles of progressors with no regard to the phase of infection.

Fig. 3 shows that EC groups 2 to 5 are clustered together and have transcription profiles different from those of untreated progressors. EC group 1 and cART-treated progressors are clustered together and have transcription profiles distinct from other groups. Thus, three large groups of distinct CD8^+^ cell transcription profiles exist: (i) EC groups 2–5, with a decreasing number of DEGs compared to healthy controls in order from 2 to 5; (ii) untreated progressors, whose profiles are different from those of ECs, which may indicate observed dysfunction of CD8^+^ lymphocytes; and (iii) EC group 1 and most of the cART-treated progressors, whose profiles are different from healthy controls but opposite to other EC groups and different from untreated progressors. We performed bootstrap resampling analysis and found that the AU and BP p-values for these three groups were equal to 1. It means that three clusters are stable under perturbations.

### 2.3 Identification of pathways and cellular processes related to identified EC groups, cART-treated and untreated progressors

**Fig. 4:**
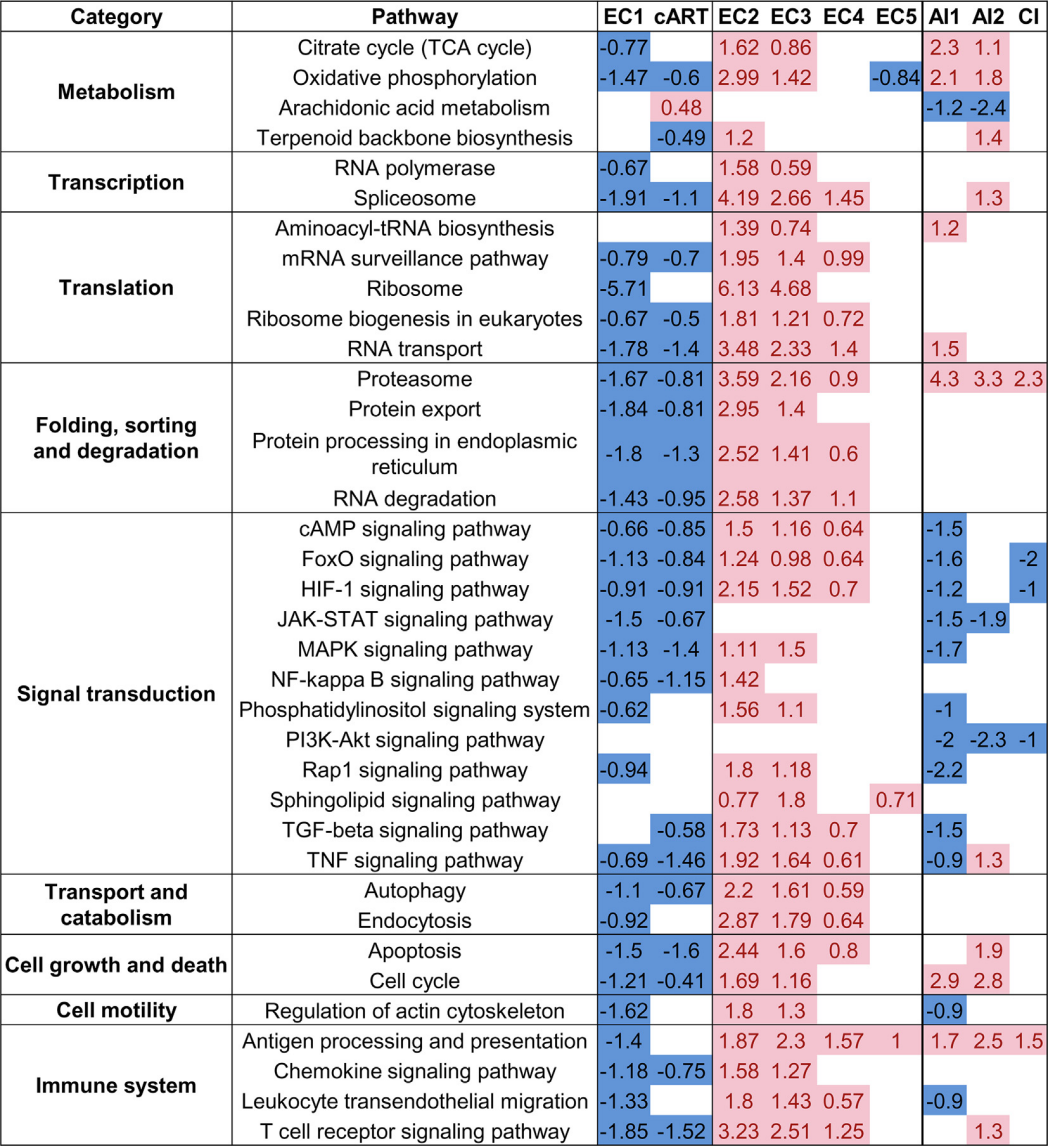
**KEGG pathways are differentially regulated in ECs, cART-treated and untreated progressors.** The values (columns 3–5) are *t*-test scores calculated in gene set enrichment analysis (see Materials and Methods). The positive value and red color mean that pathway is up-regulated, whereas the negative value and blue color mean that pathway is down-regulated compared to healthy control. EC 1–5 are groups of ECs; cART is cART-treated progressors; AI1 and AI2 are untreated progressors in the acute phase from GSE6740 and GSE25669 GEO datasets, correspondingly; CI is untreated progressors in the chronic phase from GSE6740 GEO dataset. (For interpretation of the references to color in this figure legend, the reader is referred to the web version of this article.)

To identify KEGG pathways (https://www.genome.jp/kegg/pathway.html) related to the groups mentioned above, we performed gene set enrichment analysis (see Materials and Methods). Since a particular pathway can have parts of signaling cascades, which are observed in many other pathways, and most DEGs may belong to these unspecific parts, the enrichment analysis results may contain many false-positive associations with pathways. To filter out such nonrelevant pathways, we manually checked the positions of DEGs in pathway maps. For example, if the “p53 signaling pathway” was found but the TP53 gene was not differentially expressed, the pathway was removed from further analysis. The obtained list of KEGG pathways is presented in Fig. 4. Since the involvement of pathways into HIV control, e.g., corresponding changes in functions of p53 in ECs, may not be associated with only changes in gene transcription but can be related to the changes in the proteome and post-translational modifications [29], [32] we prepared the complete list of pathways without filtering, which is presented in Table S2.

The key DEGs from pathways related to activation, survival, and immune-specific CD8^+^ lymphocyte functions are presented in Table 3. Here, we considered genes as differentially expressed if the log fold change was more than |0.5| and the unadjusted p-value was less than 0.05. The corresponding thresholds were chosen empirically to balance the number of key genes and differential expression with statistical significance.

**Table 3:** The key differentially expressed genes from revealed pathways representing the most important cellular processes, potentially related to HIV progression.

**T cell function category**	**EC group 2**–**5**	**EC group 1**	**cART-treated progressors**	**Untreated progressors**
Homing to lymph node			CCR7↓	CCR7↓, SELL↓
T cell receptor pathway	CD8A↑, ITGAL↑, CD3D↑, CD247↑, ICOS↑, LCK↓, FYN↑, PTPRC↑, NFATC2↑, NFKB1↑, JUN↑	CD8A↓, ITGAL↓, CD3D↓, CD3G↓, CD247↓, PTPRC↓, FOS↓	ICOS↓, FOS↓, JUN↓	CD8A↑, ITGAL↑, ICOS↓, FYN↑, PTPRC↓, NFKB1↓, FOS↓, JUN↑
Inhibitory immune checkpoints				PDCD1↑, CTLA4↑, LAG3↑, HAVCR2↑
Receptors for CD8^+^ lymphocytes’ growth factors	IL2RB↑, IL4R↑, IL7R↑	IL7R↓		IL4R↓, IL7R↓
Cytotoxic functions	FASLG↓, PRF1↑, GZMB↑	PRF1↓	PRF1↑	FASLG↑, PRF1↑, GZMB↑
Secreted cytokines, which compete with HIV to co-receptors	CCL3L3↑, CCL4↑, CCL5↑, CXCL12↓		CXCL12↑	CCL3↑, CCL4↑, CCL5↑
Cell cycle regulators	CCND2↑, CCNE1↓	CCND2↓		CCND2↓, CCNE1↑, CCNE2↑, CCNA2↑, CCNB1↑, CCNB2↑, CDK1↑, CDK2↑
Pro-survival genes	BCL2↑, CFLAR↑, BIRC2↑, BIRC3↑, GADD45A↑, GADD45B↑		GADD45B↓	BCL2↓, BIRC3↓, GADD45A↓, GADD45B↓
Pro-apoptotic genes	FAS↑, BAX↓, APAF1↑, DFFA↓, CASP8↑, CASP7↓, CASP2↑, CASP4↑	CASP8↓		BAK1↑, FAS↑, BAX↑, APAF1↑, BCL2L11↑, DFFA↑, CASP3↑, CASP8↑, CASP7↑, CASP4↑
Interferon-stimulated genes	ADAR↑, APOBEC3F↓, APOBEC3G↑, DDX58↓, IFITM1↑, IFNG↑, IFNGR1↑, IRF1↑, ISG15↓, JAK1↑, OAS1↓, PSMB8↑, STAT2↑, TAP1↑, TRIM5↓, TYK2↑	JAK1↓, TRIM22↓	IFNG↓, IFNGR1↓	APOBEC3F↑, APOBEC3G↑, APOBEC3H↑, BST2↑, DDX58↑, EIF2AK2↑, IFI35↑, IFI6↑, IFIH1↑, IFIT1↑, IFIT3↑, IFNG↑, IFNGR1↓, IFNGR2↓, IRF9↑, ISG15↑, JAK2↑, MX1↑, OAS1↑, OAS2↑, OAS3↑, OASL↑, PSMB8↑, PTPN2↑, SOCS1↓, STAT1↑, TAP1↑, TRIM22↑, ZBP1↑

Fig. 4 shows the same relations between EC groups, as well as treated and untreated progressors, as presented in Fig. 3. The highest number of pathways was found for ECs. Untreated progressors are associated with fewer pathways, and the direction of regulation of some of them, especially signal transduction pathways, is opposite to most ECs. The direction of pathway regulation in EC group 1 and cART-treated progressors was opposite to EC groups 2–4 as at the level of DEGs (Fig. 2, Fig. 3).

EC groups 2 and 3 are associated with the same differentially regulated pathways, whereas group 4 is associated with fewer pathways which, however, may be observed because some transcription profiles from EC group 4 are indistinguishable from healthy controls (Fig. 2), but other profiles from this group are similar to those from groups 2 and 3. The t-scores obtained for each pathway by gene set enrichment analysis (see Materials and Methods) decreased from group 2 to 4, which indicates the different magnitudes of the pathways’ differential expression. We performed gene set overrepresentation analysis (see Materials and Methods) to find KEGG pathways associated with genes that are differentially expressed in EC group 2 (EC group 3) but not differentially expressed in EC group 3 (EC group 4) (see above). The obtained pathways (Table S3) completely intersected with pathways from Fig. 4. Thus, we conclude that EC groups 2 to 4 are similar to each other at the level of pathways but different in the number of DEGs and the magnitude of fold changes. EC group 5 was indistinguishable from healthy controls at the level of pathways.

The most important cellular processes regulated by the identified pathways are as follows: (i) cell metabolism and protein synthesis; (ii) T cell activation, migration, and performing CD8^+^ cell-specific functions, including contact cytolysis of target cells and secretion of cytokines; and (iii) cell growth, proliferation, and apoptosis. The highest number of revealed pathways was related to cell metabolism and various steps of protein synthesis: from gene transcription to translation, folding, transport, and degradation (Fig. 4) (for more details see Discussion).

### 2.4 Identification of master regulators responsible for the observed transcriptional changes in ECs

Since HIV does not infect CD8^+^ lymphocytes, the observed transcription changes in ECs may be a consequence of the action of cytokines, growth factors, and mediators on corresponding receptors on the surface of cells. We used the Genome Enhancer tool (https://ge.genexplain.com) to find MRs, which are the proteins at the top of the signaling network regulating the activity of transcription factors and their complexes and, in turn, are responsible for expression changes observed in ECs (see Materials and Methods).

We selected only those MRs whose transcription changed significantly with log fold changes higher than |0.5| and p-values less than 0.5. This was done to filter out irrelevant MRs, which may not influence gene expression in ECs and may not even be expressed in CD8^+^ lymphocytes. The transcription changes of selected MRs themselves mean that they are part of positive feedback loops and are extremely important to maintain the transcription profiles observed in ECs.

**Fig. 5:**
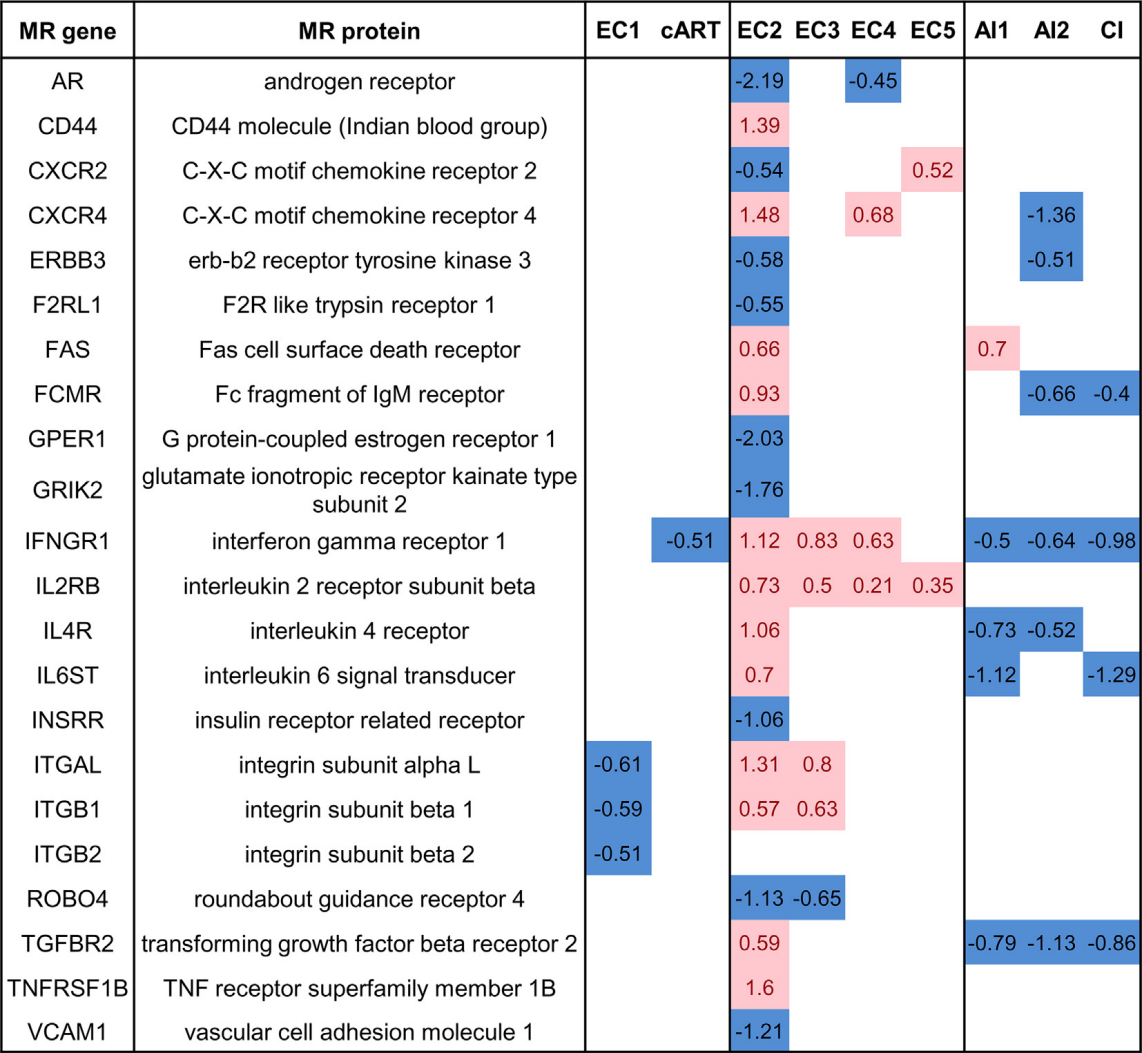
**Receptors, identified as MRs, and their transcription changes in EC groups, cART-treated and untreated progressors.** EC 1–5 are groups of ECs; cART is cART-treated progressors; AI1 and AI2 are untreated progressors in the acute phase from GSE6740 and GSE25669 GEO datasets, correspondingly; CI is untreated progressors in the chronic phase from GSE6740 GEO dataset.

We also calculated MRs for cART-treated and untreated progressors for comparison. Most MRs obtained for each group of ECs, cART-treated and untreated progressors are intracellular “hubs,” such as kinases, phosphatases, ubiquitin ligases, GTPases, and transcription factors (Table S4). We focused on receptors because their interaction with corresponding ligands is the first of the consequent events leading to gene transcription changes. As a result of the analysis, we identified 22 receptors, which may be responsible for the observed transcription changes in five EC groups (Fig. 5) (for details on receptors, see Discussion). The receptor was selected if the corresponding gene was differentially expressed with a log fold change greater than |0.5| and a p-value less than 0.5 in at least one of five EC groups.

## 3 Discussion

In our study, we performed the comprehensive analysis of the transcriptional profiles of total CD8^+^ lymphocytes (including HIV- and non-HIV-specific lymphocytes) from ECs, cART-treated, and untreated progressors using the original pipeline. The corresponding analysis allowed us to identify several groups of ECs, which are characterized by distinct transcriptional profiles, up- and down-regulated pathways, cellular processes, and MRs. Comparison of the obtained pathways, processes and MRs to those from cART-treated and untreated progressors allowed us to identify the most important differences in functional states of CD8^+^ T lymphocytes and to suggest possible mechanisms of HIV control.

We identified three large clusters (displayed in Fig. 3 and Fig. 4) of transcription profiles from the total CD8^+^ cells: (i) profiles from ECs which, in turn, formed four distinct groups (EC groups 2–5); (ii) profiles from untreated progressors, which are different from those of ECs; (iii) profiles from a small group of ECs (EC group 1) and profiles from cART-treated progressors, which are opposite to other EC groups and different from untreated progressors.

The transcriptional profiles from EC groups 2–5 differ from each other in numbers of DEGs and the magnitude of fold changes (the ratio of the average of expression values in each group to the average of expression values in healthy controls): group 2 is associated with the highest number of DEGs and highest magnitude, whereas group 5 is indistinguishable from the healthy control. Nevertheless, EC groups 2 to 4 were similar at the level of differentially regulated pathways (Fig. 4). On the other hand, these groups are different in terms of MRs (Fig. 5, Table S4), which regulate the transcription of observed DEGs. EC group 2 was associated with the highest number of differentially expressed MRs and positive feedback loops (see Results), which can explain the highest number of DEGs among all four groups (Table 2). EC groups 3 and 4 are associated with fewer and different MRs. Given the information on DEGs (Table 2), differentially expressed pathways (Fig. 4), and MRs (Fig. 5, Table S4), we can conclude that the same cellular functions are changed in CD8^+^ lymphocytes from EC groups 2–4 but with different magnitudes, and the initial causes may be different, which is reflected by different MRs.

The observed differences between EC groups may not be related to different mechanisms of HIV control but may be associated with other characteristics of patients. In accordance to study by Chowdhury and colleagues [38], the existence of EC groups 2–5 cannot be explained by differences in age, gender, race, and ethnicity of individuals, as well as the presence of protective alleles HLA-B*57/B*27/B*5801, the duration of HIV infection, viral load, the ratio of CD4^+^ / CD8^+^ cells, total CD4^+^ counts, the neutralizing breadth of HIV-specific antibodies, and the breadth and magnitude of HIV-specific cytotoxic response. At the same time, it is worth noting that the possibility of a relationship between EC groups' presence and the duration of EC status exists because it is quite challenging to assess the EC status due to the frequency of HIV load monitoring. Additionally, it is difficult to estimate the time from the beginning of infection to diagnosis. The differences between EC groups 2–5 may be explained by other unknown factors, such as the presence of different HIV variants with different impacts on CD8^+^ cell transcription and functions, co-morbidities, and changes in microbiome [32].

The identified pathways may indicate preserved functions, survival, and proliferation of CD8^+^ lymphocytes from EC groups 2–4 compared to untreated progressors. Many pathways related to metabolism and protein synthesis were up-regulated in EC groups 2–4, whereas fewer corresponding pathways were up-regulated in untreated progressors, which may indicate a lower degree of increase in metabolism (Fig. 4). The anti-apoptotic genes were up-regulated in CD8^+^ lymphocytes from EC groups 2–4 and down-regulated in untreated progressors (Table 3). The pro-apoptotic genes were upregulated in untreated progressors, whereas they have a mixed pattern of expression in EC groups 2–4. Thus, CD8^+^ lymphocytes from EC groups 2–4 have a higher ability to survive than untreated progressors; these observations are in accordance with literature data [16], [23]. The transcription of cyclins is also dissimilar in EC groups 2–4 and untreated progressors. The only gene coding cyclin D2 is up-regulated in ECs, whereas genes coding cyclins E1, E2, B1, B2, and A2 are up-regulated in untreated progressors (see Table 3). Since CD8^+^ cells from the blood are asynchronized in cell cycle phases, this observation may indicate cell cycle arrest in the G2 phase. The HIV-1 protein vpr causes G2 arrest in both infected and uninfected cells, whereas the Vif protein promotes the G1 to S phase transition [42], [43]. Only cyclins D1 and D2 are expressed in all phases of the cell cycle; thus, the presence of their transcription in EC groups 2–4 and the absence of transcription of other cyclins may indicate normal CD8^+^ lymphocyte proliferation. CD8^+^ lymphocytes from ECs are known to preserve their ability to proliferate compared to progressors [26]. The receptors of CD8^+^ cell growth factors were also up-regulated in EC groups 2–4 but down-regulated in untreated progressors (Table 3). The absence of dysfunction of CD8^+^ lymphocytes in ECs is supported by unchanged transcription of immune checkpoints, whereas the corresponding genes are up-regulated in untreated progressors [21] (Table 3). Interestingly, the transcription of genes coding perforin and granzyme B was up-regulated in both groups, but the gene coding Fas ligand was down-regulated in ECs but up-regulated in untreated progressors. CD4^+^ and CD8^+^ T lymphocytes in LTNPs have lower frequencies of apoptosis than progressors, which correlates with a lower frequency of cells expressing Fas and FasL [44]. Interferon-stimulated genes are up-regulated in untreated progressors, but mostly unchanged or even down-regulated in EC groups 2-4 compared to healthy controls. It is known that interferon activation plays a deleterious role in HIV pathogenesis, including “exhaustion” and apoptosis of lymphocytes, whereas decreased expression of interferon-stimulated genes is associated with HIV control [45], [46].

The EC group 5 is indistinguishable from a healthy control. It may indicate that total CD8^+^ lymphocytes from corresponding ECs are potentially not involved in control under viremia. On the other hand, the changes in the functional state of CD8^+^ cells from these ECs required for HIV control may not be associated with gene transcription changes but can be related to the changes in the proteome and post-translational modifications [29], [32]. Conversely, the absence of transcriptional changes in EC group 5 may indicate an exceptional control of viral replication and the absence of “bystander activation” of CD8^+^ lymphocytes [47], [48], [49], [50]. Following the study by Chowdhury and colleagues [38], all 51 elite controllers had an undetectable viral load (usually less than 50 or less than 75 copies/mL) on at least two consecutive occasions for at least two years. Viral load measured by single copy viral RNA assay and duration of HIV infection were not distinguished between EC groups 1–5. However, the blips of infection between measurements are not excluded. Thus, spontaneous cases of functional cure of HIV among studied ECs, especially from group 5, may occur. Additional extensive studies, such as assessment of HIV-DNA reservoir, are required to prove such individuals' presence. The involvement of CD8^+^ cells with unchanged gene expression in HIV control and the degree of such control should be estimated in future studies.

In contrast, the pathways associated with cART-treated progressors are differentially regulated in the opposite direction compared to ECs: they are mostly downregulated, whereas the same pathways from EC groups 2–4 are upregulated (Fig. 4). This finding may indicate the downregulation of metabolism, activation, growth, proliferation, and other essential processes in CD8^+^ T lymphocytes from cART-treated progressors. Some pathways, e.g., JAK-STAT and FoxO pathways, are changed in the same direction in cART-treated and untreated progressors (Fig. 4). However, the transcription of many important genes, e.g., pro- and anti-apoptotic genes, is not changed, unlike in untreated progressors (see Table 3). This finding may indicate that many of the considered processes are preserved in CD8^+^ T lymphocytes from cART-treated compared to untreated progressors, which is in accordance with literature data [28]. It is known that low-level viremia (~1 copy/mL of plasma) is detectable in most individuals under cART, which leads to residual activation and dysfunction of CD8^+^ T lymphocytes.

Surprisingly, EC group 1 is associated with transcription changes and pathways similar to those in cART-treated progressors. Thus, EC group 1 may also be associated with some degree of CD8^+^ T lymphocyte dysfunction and may have other viremic control mechanisms that are unrelated to CD8^+^ cells.

The observed transcription changes in CD8^+^ lymphocytes from ECs may be a consequence of the action of various cytokines, growth factors, and mediators on corresponding receptors on the surface of cells. We identified 22 receptors (Fig. 5) that were predicted as MRs by the Genome Enhancer tool and may be responsible for the expression changes observed in ECs. Many of the identified receptors are expressed in the opposite direction in ECs and untreated progressors. Taking into account the key roles that these receptors play in gene expression changes in ECs and progressors, they may represent the potential targets of therapeutic intervention, which enables switching of the phenotype of CD8^+^ lymphocytes to those in ECs, decelerating disease progression and potentially increasing the antiviral noncytotoxic response.

Most of the identified receptors are related to various cytokines, which are important for regulating CD8^+^ lymphocyte functions. For instance, the interferon-gamma receptor is a potential MR for DEGs from both ECs and treated and untreated progressors. IFNGR1 gene transcription changed in the opposite direction in ECs and progressors. Interferon-gamma is one of the most important cytokines required to increase CD8^+^ lymphocyte abundance during viral infection and regulate their homeostasis [51], [52]. The IL-4 receptor gene is unexpectedly upregulated in EC group 2 and downregulated in untreated progressors. IL-4 is a T helper 2 cytokine that reduces the ability of CD8^+^ lymphocytes to suppress HIV infection [53]. Gene IL2RB coding subunit beta of the IL-2 receptor is upregulated in ECs but unchanged in progressors. In contrast to IL-4, IL-2 enhances CD8^+^ T cell anti-HIV activity and optimizes both effector T cell generation and differentiation into memory cells [53], [54]. Thus, it appears that the combination of signaling from various receptors, rather than from a single receptor, may cause gene expression changes and functional states observed in ECs and progressors.

Some of the identified receptors, e.g., androgen and G-coupled estrogen receptors, are not typically associated with CD8^+^ lymphocytes; however, they may also contribute to expression changes in ECs. For instance, the androgen receptor gene is downregulated in EC groups 2 and 4, but its expression is not changed in progressors. It was shown that women are significantly overrepresented in the EC population compared to men [26]. This finding can be explained by influence of androgens on the immune system, including CD8^+^ lymphocytes [55]. Thus, the androgen receptor may play a significant role in the observed phenomenon.

## 4 Conclusions

We have developed an original pipeline to analyze the transcription profiles of CD8^+^ lymphocytes from a large cohort of ECs, cART-treated and untreated progressors. This pipeline includes cluster analysis of CD8^+^ cells’ transcription profiles from ECs, comparison of the obtained clusters to corresponding profiles from cART-treated and untreated progressors, identification and comparison of pathways and cellular processes between revealed groups of ECs and progressors, and identification of receptors, which are potential MRs and may be responsible for observed gene expression changes in EC groups. The application of the pipeline allowed us to obtain the new findings regarding the heterogeneity of the mechanisms of HIV control in ECs.

First, we identified five EC groups (EC groups 1–5) with distinct transcription profiles from CD8^+^ lymphocytes. Second, we analyzed the peculiarities of each group of ECs associated with their transcription profiles. Particularly, the transcriptional profiles of EC group 1 were opposite to those of EC groups 2–4 and similar to those of the cART-treated progressors. The transcriptional profiles of EC groups 2–4 are distinguishable from healthy controls and similar to each other in terms of DEGs and pathways, but they differ in the magnitude of gene expression changes so that the number of DEGs and magnitude increase from EC group 4 to group 2. The EC groups 2–4 are also different from each other at the level of potential MRs, which may indicate the different original causes of observed transcription changes. The transcription profiles from EC group 5 are indistinguishable from healthy controls.

Comparison of transcriptional profiles between five groups of ECs and both untreated and cART-treated progressors allowed us to identify key pathways, processes, and MRs, which may be essential for HIV control and may represent potential therapeutic intervention targets. We demonstrated that transcription changes in EC groups 2–4 are distinct from untreated progressors and opposite to cART-treated progressors. According to pathway analysis, the CD8^+^ lymphocytes from EC groups 2–4 associated with increased survival, proliferation, cellular metabolism, and protein synthesis compared to cells from both untreated progressors and uninfected individuals. Compared to untreated progressors, the CD8^+^ lymphocytes from EC groups 2–4 are not associated with the “exhausted” phenotype: a state of CD8^+^ cells characterized by an increased expression of inhibitory immune checkpoints (PD-1, Lag-3, and Tim-3) on the cell surface leading to a decreased ability of CD8^+^ cells to proliferate, secrete cytokines and induce cytotoxic effect. These findings may explain the observed viral control status of ECs from groups 2–4 and indicate the potential involvement of non-HIV-specific CD8^+^ lymphocytes in noncytotoxic antiviral functions.

The corresponding profiles from EC group 1 are opposite to those of EC groups 2–4 and similar to those of the cART-treated progressors. The ECs from group 1 may be associated with low-level viremia, which is present in cART-treated progressors, that leads to residual activation and dysfunction of CD8^+^ T lymphocytes. According to the findings mentioned above, EC groups 1 and 5 may have mechanisms of HIV control unrelated to transcriptional changes in CD8^+^ cells.

Finally, we identified 22 receptors, whose modulation may be responsible for the observed transcription changes and functional state of CD8^+^ lymphocytes from ECs. Besides receptors for cytokines, hormones and mediators receptors were identified, e.g., androgen receptor and G-coupled estrogen receptors, whose relationships with ECs were not previously described. The directions of changes in the transcription of receptor-coding genes are similar or opposite to untreated progressors depending on the receptor. Thus, it appears that the combination of signals from various receptors, rather than from a single receptor, may cause gene expression changes and functional states observed in ECs. The revealed receptors, especially those whose transcription changes are different in ECs and untreated progressors, e.g., interferon-gamma or androgen receptors, and their combinations may represent the potential targets of therapeutic intervention, enabling switching the phenotype of CD8^+^ lymphocytes to those in ECs, decelerating disease progression, and potentially increasing the antiviral responses.

The pipeline developed can be further applied for a comprehensive analysis of the transcription profiles of some other viral infectious diseases to find potential host targets for the development of novel antiviral medicines.

## 5 Materials and Methods

### 5.1 Transcriptional datasets

The three datasets GSE87620, GSE6740, and GSE25669 with CD8^+^ cell transcriptional profiles from ECs, cART-treated and untreated progressors, and uninfected individuals were obtained from Gene Expression Omnibus (GEO) (https://www.ncbi.nlm.nih.gov/geo). Despite some other HIV- and EC-related transcriptomics experiments were done in recent years and corresponding data was publicly available [32], only these three datasets contained samples of CD8^+^ lymphocytes from healthy uninfected individuals, which was important for the performed analysis. The dataset GSE87620 includes 51 samples from ECs, 32 samples from cART-treated patients, and 10 samples from uninfected people. The dataset GSE6740 contains data on CD8^+^ lymphocytes from untreated progressors in acute (5 samples) and chronic (5 samples) phases of infection and uninfected individuals (5 samples). The dataset GSE25669 contains corresponding information from untreated progressors in the acute phase (4 samples) and uninfected people (2 samples). The transcriptional profiles from GSE25669, GSE87620, and GSE6740 datasets were measured on three microarray platforms: Illumina HumanHT-12 V3.0 and V4.0 expression bead chips and Affymetrix Human Genome U133A Array, correspondingly.

### 5.2 Preprocessing, clustering of samples and identification of differentially expressed genes

The corresponding analysis was performed using various R packages from The Comprehensive R Archive Network (https://cran.r-project.org) and Bioconductor (https://www.bioconductor.org). All steps of analysis were performed separately on each of the three datasets.

Background correction and quantile normalization of transcription data were performed using different functions depending on the microarray platform: function “rma” from the “affy” package for Affymetrix microarray (GSE6740 dataset) and the “neqc” function from the “limma” package for Illumina bead chips (GSE87620 and GSE25669 datasets).

Next, we removed probes that were unexpressed in all samples of the dataset. To filter out corresponding probes on the Affymetrix microarray, we used the “mas5calls” function from the “affy” package and removed probes having an “absent” score across all samples. To do this on Illumina bead chips, we removed probes that have detection p-values greater than 0.05 in all samples of the dataset.

We selected only probes having Entrez IDs and only one probe per gene with the highest variance among samples using the “nsFilter” function from the “genefilter” package. It was done because of three reasons. First, the probes with the highest variance are the most informative for identifying differences between elite controllers' transcription profiles since they have different expression values in different profiles. Second, most applied methods required transcription data at the level of genes but not probes, e.g., gene set enrichment analysis and search for MRs. Third, comparing DEGs and MRs, between transcription datasets derived from different microarray platforms also required gene-level transcription data. The obtained gene transcription profiles were used at all stages of the pipeline.

To find potential clusters on heterogenic transcriptional profiles from ECs, we selected 51 corresponding samples and filtered out 50 percent of genes with the lowest variance using the “nsFilter” function. This step was employed to remove genes whose expression is not changed significantly across samples and cannot be useful to find potential EC groups. To find clusters, we used a hierarchical agglomerative clustering approach implemented in the “hclust” basic R function. We choose 1 – Pearson correlation coefficient between pairs of samples as a distance measure, and the “ward.D2” clustering method [56]. To estimate the uncertainty in obtained clusters, we performed multiscale bootstrap resampling using the “pvclust” function from the “pvclust” package. For each cluster, “pvclust” calculates p-values, which indicate how strongly the cluster is supported by data: the higher the p-value, the more probable the cluster's existence. Function “pvclust” provides two types of p-values: the AU (Approximately Unbiased) p-value and BP (Bootstrap Probability) value. The AU p-value is computed by multiscale bootstrap resampling, whereas the BP value is computed by normal bootstrap resampling [57].

To visualize clusters, we used the “heatmap.2” function from the “gplots” R package. This function creates a heatmap with two dendrograms: one for rows, which are genes, and another for columns, which are samples. To create these dendrograms, the abovementioned clustering method (“ward.D2”) and distance measure (1 – Pearson correlation coefficient) were used. The lengths of dendrogram branches are proportional to 1 – Pearson correlation coefficient values. To highlight the differences in gene expression values between samples, row Z-scores were calculated. The row Z-score is the number of standard deviations by which the value of gene expression in a particular sample is above or below the mean value of all samples.

To identify DEGs for each of the obtained clusters of ECs, groups of cART-treated and untreated progressors compared to uninfected people, we used the Linear Models for Microarray Data (Limma) approach [58]. The analysis was performed using functions from the “limma” R package. The thresholds on log fold changes and Benjamini-Hochberg corrected (adjusted) p-values were chosen depending on the particular analysis.

### 5.3 Gene set enrichment analysis

To identify KEGG pathways (https://www.genome.jp/kegg/pathway.html) that were differentially regulated in each group of ECs, as well as in cART-treated and untreated progressors compared to uninfected persons, we performed gene set enrichment analysis [59] using the “gage” function from the “gage” R package [60]. Function “gage” implemented a two-sample *t*-test for determining the differential expression of genesets, e.g., genes from a particular pathway, between two conditions, e.g., samples from cART-treated progressors and uninfected individuals. We selected pathways with an adjusted p-value of less than 0.1, which is the default threshold. The required data on relations between human genes and KEGG pathways were retrieved from the Enrichr database (https://amp.pharm.mssm.edu/Enrichr/#stats).

### 5.4 Gene set overrepresentation analysis

To identify KEGG pathways associated with genes that are differentially expressed in EC group 2 (EC group 3) but not differentially expressed in EC group 3 (EC group 4), gene set overrepresentation analysis was used [59]. This analysis allows the identification of pathways where investigated genes are overrepresented compared to the background gene set, e.g., all genome genes. To perform analysis, we used the function “enrichr” from the “enrichR” R package. We selected pathways with an adjusted p-value less than 0.1, as in the gene set enrichment analysis.

### 5.5 Identification of master regulators

To identify MRs, we used the Genome Enhancer tool (https://ge.genexplain.com) developed by geneXplain GmbH [66]. Briefly, Genome Enhancer implemented a pipeline including three main steps: (i) analyze promoter regions of genes to predict transcription factor binding sites using positional weight matrices from the TRANSFAC database [61]; (ii) since it is clear by now that combinations of TFs, rather than a single TF, drive gene transcription and define its specificity, the combinations of TF binding sites called “composite regulatory modules” are identified [62]; and (iii) reconstruct the signaling pathways that activate these TFs and identify master regulators at the top of such pathways [63], [64]. This analysis uses a signaling network from the TRANSPATH database [65].

### 5.6 Implementation of the pipeline in R

The R scripts implementing all steps of the pipeline, except the search for MRs realizing in Genome Enhancer tool (https://ge.genexplain.com), are available on the GitHub repository (https://github.com/serivanov86/EC_transcription_analysis).

## CRediT authorship contribution statement

**Sergey Ivanov:** Conceptualization, Data curation, Methodology, Software, Writing - original draft, Writing - review & editing. **Dmitry Filimonov:** Methodology, Software, Writing - review & editing. **Olga Tarasova:** Conceptualization, Data curation, Methodology, Supervision, Writing - original draft, Writing - review & editing.

## Declaration of Competing Interest

The authors declare that they have no known competing financial interests or personal relationships that could have appeared to influence the work reported in this paper.
